# High Prevalence of Severe Food Insecurity and Malnutrition among HIV-Infected Adults in Senegal, West Africa

**DOI:** 10.1371/journal.pone.0141819

**Published:** 2015-11-03

**Authors:** Noelle A. Benzekri, Jacques Sambou, Binetou Diaw, El Hadji Ibrahima Sall, Fatima Sall, Alassane Niang, Selly Ba, Ndèye Fatou Ngom Guèye, Mouhamadou Baïla Diallo, Stephen E. Hawes, Moussa Seydi, Geoffrey S. Gottlieb

**Affiliations:** 1 Department of Medicine, University of Washington, Seattle, WA, United States of America; 2 Centre de Santé de Ziguinchor, Ziguinchor, Sénégal; 3 Centre Hospitalier Universitaire de Fann, Dakar, Sénégal; 4 Department of Global Health, University of Washington, Seattle, WA, United States of America; 5 Department of Epidemiology, University of Washington, Seattle, WA, United States of America; Rush University, UNITED STATES

## Abstract

**Background:**

Malnutrition and food insecurity are associated with increased mortality and poor clinical outcomes among people living with HIV/AIDS; however, the prevalence of malnutrition and food insecurity among people living with HIV/AIDS in Senegal, West Africa is unknown. The objective of this study was to determine the prevalence and severity of food insecurity and malnutrition among HIV-infected adults in Senegal, and to identify associations between food insecurity, malnutrition, and HIV outcomes.

**Methods:**

We conducted a cross-sectional study at outpatient clinics in Dakar and Ziguinchor, Senegal. Data were collected using participant interviews, anthropometry, the Household Food Insecurity Access Scale, the Individual Dietary Diversity Scale, and chart review.

**Results:**

One hundred and nine HIV-1 and/or HIV-2 participants were enrolled. The prevalence of food insecurity was 84.6% in Dakar and 89.5% in Ziguinchor. The prevalence of severe food insecurity was 59.6% in Dakar and 75.4% in Ziguinchor. The prevalence of malnutrition (BMI <18.5) was 19.2% in Dakar and 26.3% in Ziguinchor. Severe food insecurity was associated with missing clinic appointments (p = 0.01) and not taking antiretroviral therapy due to hunger (p = 0.02). Malnutrition was associated with lower CD4 cell counts (p = 0.01).

**Conclusions:**

Severe food insecurity and malnutrition are highly prevalent among HIV-infected adults in both Dakar and Ziguinchor, and are associated with poor HIV outcomes. Our findings warrant further studies to determine the root causes of malnutrition and food insecurity in Senegal, and the short- and long-term impacts of malnutrition and food insecurity on HIV care. Urgent interventions are needed to address the unacceptably high rates of malnutrition and food insecurity in this population.

## Introduction

Malnutrition is associated with increased mortality among individuals starting antiretroviral therapy (ART) [1–11]. Food insecurity, defined as a lack of access to sufficient, safe, nutritious food to meet dietary needs and maintain a healthy and active life [12], is associated with poor adherence to ART [13–15] which can lead to virologic failure, impaired immunological recovery, and death. Understanding the relationships between HIV, malnutrition, and food insecurity has important implications for the effective integration of nutritional interventions into HIV programs. This is of particular importance in sub-Saharan Africa, which is home to 70% of the ~35 million people living with HIV [16] and a quarter of the 805 million people worldwide who are undernourished [17].

As a consequence of climate change, conflict, and economic instability, the increasing burdens of food insecurity and malnutrition in West Africa represent a critical global health challenge [18]. However, there have been no published studies examining the relationships between HIV, food insecurity, and malnutrition in this region.

In Dakar, the capital of Senegal, the predominant source of income is non-agricultural business and wages, while in Ziguinchor, located in the southern Casamance region of the country, it is agriculture [19]. Although the economy in Casamance is based on agriculture, the region has been involved in a longstanding civil conflict, and has the highest rates of chronic malnutrition and food insecurity in the country [20]. The prevalence of food insecurity among the general population in Senegal is 16%, while in certain communities of the Casamance the prevalence exceeds 60%. Based on estimates among children in the general population, the prevalence of chronic malnutrition is 16.5%, with a prevalence as high as 31.0% in parts of the Casamance. The Casamance has also been the most severely affected by the HIV epidemic in Senegal. The prevalence of HIV in Ziguinchor is 1.3–4.0%, while the prevalence in Dakar is 0.8–2.7% [21]. ART is provided free of charge through the Initiative Sénégalaise d'accès aux Antirétroviraux (ISAARV). Current criteria for ART initiation are CD4 count ≤ 500 cell/mm^3^ and/or WHO Stage 3 or 4 disease. Despite the high prevalence of food insecurity and malnutrition among the general population in Senegal, the prevalence of food insecurity and malnutrition among individuals infected with HIV is unknown.

The objective of this study was to determine the prevalence and severity of food insecurity and malnutrition among HIV-infected adults in Senegal, West Africa and to identify associations between food insecurity, malnutrition, and HIV outcomes.

## Methods

We conducted a cross-sectional study from February to March 2015 in Dakar and Ziguinchor, Senegal. The study took place at the Centre Hospitalier Universitaire de Fann, located in Dakar and the Centre de Santé de Ziguinchor, located in Ziguinchor. HIV-infected individuals ≥18 years of age who provided written informed consent were eligible for participation. Individuals who were pregnant, breastfeeding, or enrolled in other research studies were excluded. Study procedures were approved by the University of Washington Institutional Review Board and the Senegal Comité National d'Ethique pour la Recherche en Santé.

The study encounter was conducted in the participant’s preferred language, including French, Wolof, Peular, Diola, Mandinka, or Creole. Participants completed interviewer-administered questionnaires and participated in semi-structured interviews to determine participant socioeconomic characteristics, household economic indicators, and behaviors. We used the Household Food Insecurity Access Scale (HFIAS) [22] to determine food insecurity status and the Individual Dietary Diversity Scale (IDDS) [23] to determine dietary diversity. The HFIAS was developed by the USAID Food and Nutrition Technical Assistance (FANTA) project to differentiate food secure from food insecure households across different cultural contexts. It is a 9-item questionnaire which provides a food insecurity score on a scale of 1–4, with 1 being not food insecure, 2 being mildly food insecure, 3 being moderately food insecure, and 4 being severely food insecure. A HFIAS score of 2–4 was considered food insecure and a HFIAS of 4 was considered severely food insecure.

The IDDS was developed by the Food and Agriculture Organization of the United Nations. It provides a dietary diversity score based on food intake in the past 24 hours. Foods are categorized into 12 food groups: cereals, white roots/tubers, vegetables, fruits, meat, eggs, fish/seafood, legumes/nuts/seeds, dairy, oil/fats, sweets, and spices/condiments/beverages. The IDDS score ranges from 0 to 12 with higher values representing greater dietary diversity.

Participant height, weight, and mid-upper arm circumference (MUAC) were measured. Our measure of malnutrition was a Body Mass Index (BMI) (weight (kg) / [height (m)]^2^) of <18.5. A BMI <15.00 was considered very severely underweight, 15.00–15.99 was considered severely underweight, 16.00–18.49 was considered underweight, 18.50–24.99 was considered normal weight, 25.00–29.99 was considered overweight, and 30.00 was considered obese. Mid-upper arm circumference was classified as ≤230mm or >230mm [24].

Medical records were reviewed to determine HIV type (HIV-1, HIV-2, or dual infection), WHO clinical stage, CD4 count, date of HIV diagnosis, history of antiretroviral therapy (ART), ART regimen, and use of co-trimoxazole. When date of diagnosis was not available in the medical record, patient reported dates were used. The most recent CD4 count available in the medical record was used for analysis. Adherence to clinic appointments and ART was determined by patient report. Patients responded “yes” or “no” to questions exploring potential reasons for missing clinic appointments or ART doses. In addition, they were also asked to quantify the number of days in which they failed to take their ART in the past 7 days.

Data were analyzed using SPSS Statistics 19 [IBM]. Descriptive analysis was performed for all variables. Chi-square and Fisher’s Exact tests were used to identify differences between groups. Raw data that were not normally distributed were transformed using log10 or square root transformation. The t-test was used to identify differences in means between groups. Linear regression was used to identify variables associated with BMI. Missing data were excluded from analysis. P-values <0.05 were considered significant.

## Results

In total, 109 individuals participated in this study, 52 (48%) in Dakar and 57 (52%) in Ziguinchor (Table 1). Ages ranged from 19–67 years with a mean age of 45 years in Dakar and 43 years in Ziguinchor. The majority were female. The majority (>90%) were of Senegalese nationality, with the remainder coming from Guinea-Bissau, Guinea-Conakry, Gabon, Gambia, and Mali. Most lived locally, in the Dakar region or in the town of Ziguinchor. Participants in Dakar spent more than twice as much time traveling to clinic than participants in Ziguinchor (122 minutes versus 51 minutes). There were more participants who were never married in Ziguinchor and more widows in Dakar. Educational level did not differ between sites; slightly less than 30% of participants had completed primary school. Approximately 60% of participants in Dakar were unemployed versus 45.6% in Ziguinchor. In both sites, the majority of participants owned personal cell-phones. Household size ranged from 1–40 individuals per household, with approximately one third living in households of 11 individuals or more. In Ziguinchor fewer participants had household electricity, televisions, or piped water, and more had simple latrines. In Dakar more individuals had tile floors, whereas in Ziguinchor more had earthen floors of dirt or sand.

**Table 1:** Participant characteristics in Dakar and Ziguinchor, Senegal, West Africa.

	Both sites n (%)	Dakar n (%)	Ziguinchor n (%)	p-value
**Number of Participants (N)**	109 (100)	52 (48)	57 (52)	
**Age (years), mean (range)**	44 (19–67)	45 (20–61)	43 (19–67)	0.34
**Female**	91 (83.5)	47 (90.4)	44 (77.2)	0.08
**Senegalese** ^a^ **(nationality)**	99 (90.8)	47 (90.4)	52 (91.2)	1.00
**Local residence** ^b^	85 (78.0)	43 (82.7)	42 (73.7)	0.36
**Mean transport time to clinic (minutes)**	**85**	**122**	**51**	**0.01** *
**Marital status**				**0.04**
** Never married**	**13 (11.9)**	**2 (3.8)**	**11 (19.3)**	**-**
** Monogamous**	**26 (23.9)**	**10 (19.2)**	**16 (28.1)**	**-**
** Polygamous**	**17 (15.6)**	**8 (15.4)**	**9 (15.8)**	**-**
** Divorced**	**15 (13.8)**	**8 (15.4)**	**7 (12.3)**	**-**
** Widowed**	**38 (34.9)**	**24 (46.2)**	**14 (24.6)**	**-**
**Educated** ^c^	31 (28.4)	15 (28.8)	16 (28.1)	1.00
**Unemployed**	57 (52.3)	31 (59.6)	26 (45.6)	0.18
**Personal cell-phone**	86 (79.6)	39 (75.0)	47 (82.5)	0.48
**Household (HH) size** ^d^				0.45
** 1–5**	31 (28.4)	17 (32.7)	14 (24.6)	-
** 6–10**	43 (39.4)	19 (36.5)	24 (42.1)	-
** 11–15**	14 (12.8)	8 (15.4)	6 (10.5)	-
** 16–20**	12 (11.0)	6 (11.5)	6 (10.5)	-
** 21–40**	9 (8.3)	2 (3.8)	7 (12.3)	-
**HH monthly income (USD)** ^e^ **, mean (range)**	134.1 (0–670.4)	157.2 (0–670.4)	114.4 (0–593.7)	0.54**
**Economic Indicators**				
** HH Electricity**	**80 (74.1)**	**44 (86.3)**	**36 (63.2)**	**0.01**
** HH Television**	**70 (64.8)**	**41 (80.4)**	**29 (50.9)**	**0.01**
** HH Water, piped**	**68 (63.0)**	**45 (88.2)**	**23 (40.4)**	**0.01**
** HH Toilet**				**0.01**
** Simple latrine**	**31 (28.7)**	**5 (9.8)**	**26 (45.6)**	-
** Latrine w/ slab**	**56 (51.9)**	**28 (54.9)**	**28 (49.1)**	-
** Flush toilet**	**21 (19.4)**	**18 (35.3)**	**3 (5.3)**	-
** HH floor**				**0.01**
** Earthen**	**31 (28.7)**	**7 (13.7)**	**24 (42.1)**	-
** Cement**	**40 (37.0)**	**12 (23.5)**	**28 (49.1)**	-
** Tile**	**37 (34.3)**	**32 (62.7)**	**5 (8.8)**	-
**HH Livestock**	**40 (36.7)**	**12 (23.1)**	**28 (49.1)**	**0.01**
**HH Agriculture**	**20 (18.3)**	**3 (5.8)**	**17 (29.8)**	**0.01**
**HH daily food expenditure (USD)** ^f^ **, mean (range)**	**3.5 (0–14.5)**	**4.7 (0–14.5)**	**2.2 (0–8.1)**	**0.01** **
**Ever received food aid**	42 (38.5)	15 (28.8)	27 (47.4)	0.05
**Years since HIV diagnosis, mean (range)**	**5.5 (0.0–21.2)**	**7.4 (0.1–21.2)**	**3.5 (0.0–13.3)**	**0.01** *
**HIV type**				0.20
** 1**	80 (78.4)	43 (84.3)	37 (72.5)	-
** 2**	20 (19.6)	8 (15.7)	12 (23.5)	-
** 1 and 2**	2 (1.9)	0	2 (3.9)	-
**WHO Stage 3 or 4**	51 (54.3)	25 (55.6)	26 (53.1)	0.84
**Mean CD4**	**426**	**502**	**354**	**0.01**
**On ART**	97 (89.0)	44 (84.6)	53 (93.0)	0.22
**Years on ART, mean (range)**	3.6 (0.1–16.0)	4.5 (0.2–16.0)	2.7 (0.1–10.7)	0.11*
**Number of different ART regimens**				0.57
** 1**	61 (67.8)	27 (65.9)	34 (69.4)	-
** 2**	18 (20.0)	10 (24.4)	8 (16.3)	-
** ≥3**	11 (12.2)	4 (9.8)	7 (14.3)	-
**Current protease inhibitor-based regimen**	20 (22.2)	6 (14.6)	14 (28.6)	0.13
**Current zidovudine (AZT)-use**	34 (37.8)	13 (31.7)	21 (42.9)	0.38
**Current co-trimoxazole prophylaxis**	60 (62.5)	26 (53.1)	34 (70.8)	0.06
**Missed any ART in the past 7 days**	20 (20.8)	7 (15.9)	13 (25.0)	0.32

^a^Non-Senegalese nationalities = Guinea-Bissau, Guinea-Conakry, Gabon, Gambia, Mali

^b^Residence in Dakar Region or Ziguinchor town

^c^Educated is defined as completed primary school or more

^d^Number of individuals per household

^e^Estimated household monthly income in U.S. Dollars

^f^Estimated U.S. Dollars spent on food for the household per day

*Mean and range based on raw data, p-value based on log10 transformation

**Mean and range based on raw data, p-value based on square root transformation

In Ziguinchor, more participants owned livestock (p = 0.01) or practiced agriculture (p = 0.01) compared to Dakar. In Ziguinchor, 49.1% of participants owned livestock and 29.8% participated in agriculture, whereas in Dakar 23.1% of participants owned livestock and only 5.8% participated in agriculture. Household expenditure on daily food was greater in Dakar ($4.70) compared to Ziguinchor ($2.20) (p = 0.01). In Ziguinchor, 47.4% of participants had a history of receiving food aid, compared to 28.8% in Dakar (p = 0.05). However, none of the participants were receiving food aid at the time of the study encounter.

The number of years since HIV diagnosis ranged from 0–21.2 with a mean number of years since diagnosis in Dakar of 7.4 compared to 3.5 in Ziguinchor (p = 0.01) (Table 1). The majority of participants in both sites were infected with HIV-1. In Ziguinchor, 23.5% of individuals were infected with HIV-2 and 3.9% were dually infected, versus 15.7% infected with HIV-2 in Dakar. More than half of participants had WHO stage 3 or 4 disease. The mean CD4 count in Dakar was 502 versus 354 in Ziguinchor (p = 0.01). In both sites, the majority of participants were receiving ART (84.6% in Dakar and 93.0% in Ziguinchor) and the majority (67.8%) had been on one ART regimen. Among all participants, 22.2% were receiving a protease inhibitor-based regimen, 37.8% were receiving AZT as part of their ART regimen, and 62.5% were receiving co-trimoxazole. In Ziguinchor, 25.0% of participants reported missing at least one day of ART in the past seven days versus 15.9% in Dakar (p = 0.32).

The prevalence of malnutrition (BMI <18.5) was 19.2% in Dakar and 26.3% in Ziguinchor (p = 0.50) (Table 2). More than a fifth of participants had a MUAC ≤230mm. The prevalence of food insecurity was 84.6% in Dakar and 89.5% in Ziguinchor (p = 0.57). The prevalence of severe food insecurity was 59.6% in Dakar and 75.4% in Ziguinchor (p = 0.10). Mean Individual Dietary Diversity Scale (IDDS) scores were 6.5 in Dakar and 5.9 in Ziguinchor (p = 0.10).

**Table 2:** Nutritional status, food security status, and behaviors of participants in Dakar (n = 52) and Ziguinchor (n = 57), Senegal.

	Both sites n (%)	Dakar n (%)	Ziguinchor n (%)	p-value
**BMI, mean (range)**	23.4 (11.6–38.7)	23.5 (11.6–38.7)	23.3 (11.9–36.1)	0.92
**Malnourished (BMI <18.5)**	25 (22.9)	10 (19.2)	15 (26.3)	0.50
**BMI categories**				0.88*
** Very severely underweight (BMI <15.00)**	4 (3.7)	2 (3.8)	2 (3.5)	-
** Severely underweight (BMI 15.00–15.99)**	0 (0)	0 (0)	0 (0)	-
** Underweight (BMI 16.00–18.49)**	21 (19.3)	8 (15.4)	13 (22.8)	-
** Normal weight (BMI 18.50–24.99)**	45 (41.3)	22 (42.3)	23 (40.4)	-
** Overweight (BMI 25.00–29.99)**	24 (22.0)	13 (25.0)	11 (19.3)	-
** Obese (BMI ≥30.00)**	15 (13.8)	7 (13.5)	8 (14.0)	-
**MUAC (mm), mean (range)**	270 (145–385)	269 (150–385)	271 (145–385)	0.87
** MUAC ≤ 230mm**	24 (22.2)	12 (23.1)	12 (21.4)	1.00
**Food Insecure** ^a^	95 (87.2)	44 (84.6)	51 (89.5)	0.57
**Severely Food Insecure** ^a^	74 (67.9)	31 (59.6)	43 (75.4)	0.10
**IDDS score, mean (range)**	6.2 (0–10)	6.5 (0–10)	5.9 (0–10)	0.10
**Decrease meal size due to food insecurity**	71 (65.1)	32 (61.5)	39 (68.4)	0.55
**Skip meals due to food insecurity**	51 (46.8)	22 (42.3)	29 (50.9)	0.44
**Remain 24 hours without eating due to food insecurity**	14 (12.8)	5 (9.6)	9 (15.8)	0.40
**Miss appointments due to hunger**	12 (11.4)	9 (17.3)	3 (5.7)	0.07
**Don’t take ART due to hunger**	16 (16.7)	4 (9.1)	12 (23.1)	0.10
**Miss meals due to ART effects**	**59 (61.5)**	**18 (40.9)**	**41 (78.8)**	**0.01**

BMI = Body Mass Index; MUAC = Mid-upper arm circumference; IDDS = Individual Dietary Diversity Scale (see text)

^a^Food Insecure: HFIAS score = 2–4, Severely Food Insecure: HFIAS score = 4

*p-value for trend

The majority of participants decreased meal size in the previous 4 weeks as a result of food insecurity (Table 2). In Dakar 42.3% reported skipping meals versus approximately 51.0% in Ziguinchor. Approximately 10.0% of individuals in Dakar remained 24 hours without eating during the previous 4 weeks due to food insecurity and approximately 16.0% remained 24 hours without eating in Ziguinchor.

In Dakar, 17.3% miss clinic appointments due to hunger, versus 5.7% in Ziguinchor. However, in Ziguinchor 23.1% don’t take their ART due to hunger, versus 9.1% in Dakar (Table 2). Missing meals due to ART adverse effects was more common in Ziguinchor. Missing meals due to ART adverse effects was additionally associated with being on a protease inhibitor-based regimen (p = 0.03).

When data for participants in Dakar and Ziguinchor were combined, the majority of participants were severely food insecure (Table 3). Severe food insecurity was not associated with BMI or malnutrition. A MUAC ≤230mm was less common among those who were severely food insecure compared to those who were not severely food insecure. Severe food insecurity was associated with lower dietary diversity, less education, all economic indicators indicative of lower socioeconomic status, lower daily expenditure on household food, and a history of receiving food aid. There was a trend towards smaller household size among those who were not severely food insecure (p = 0.07). Severe food insecurity was not associated with livestock ownership or household agriculture.

**Table 3:** Comparison of participant characteristics according to food security status^a^.

	Severely Food Insecure (%)	Not Severely Food Insecure (%)	p-value
**N**	74	35	-
**BMI, mean (range)**	23.5 (11.6–38.7)	23.3 (13.0–36.0)	0.88
**Malnourished (BMI <18.5)**	16 (21.6)	9 (25.7)	0.63
**BMI categories**			0.86*
** Very severely underweight (BMI <15.00)**	2 (2.7)	2 (5.7)	-
** Severely underweight (BMI 15.00–15.99)**	0 (0)	0 (0)	-
** Underweight (BMI 16.00–18.49)**	14 (18.9)	7 (20.0)	-
** Normal weight (BMI 18.50–24.99)**	32 (43.2)	13 (37.1)	-
** Overweight (BMI 25.00–29.99)**	17 (23.0)	7 (20.0)	-
** Obese (BMI ≥30.00)**	9 (12.2)	6 (17.1)	-
**MUAC, mean (range)**	273.5 (145–385)	261.8 (170–330)	0.24
** MUAC ≤ 230mm**	**12 (16.2)**	**12 (35.3)**	**0.04**
**IDDS score, mean**	**5.8**	**7.1**	**0.01**
**Age (years), mean (range)**	44.8 (19–67)	40.9 (20–60)	0.07
**Female**	62 (83.8)	29 (82.9)	1.00
**Marital status- Never married**	6 (8.1)	7 (20.0)	0.11
**Marital status- Widowed**	25 (33.8)	13 (37.1)	0.83
**Educated** ^b^	**16 (21.6)**	**15 (42.9)**	**0.04**
**Unemployed**	41 (55.4)	16 (45.7)	0.41
**Personal cell-phone**	58 (78.4)	28 (82.4)	0.80
**HH size** ^c^ **≤ 5**	17 (23.0)	14 (40.0)	0.07
**HH size** ^c^ **≤ 10**	52 (70.3)	22 (62.9)	0.51
**HH monthly income (USD)** ^d^ **, mean (range)**	**99.2 (0–593.7)**	**222.9 (0–670.4)**	**0.01** **
**Economic Indicators**			
** HH Electricity**	**49 (66.2)**	**31 (91.2)**	**0.01**
** HH Television**	**40 (54.1)**	**30 (88.2)**	**0.01**
** HH Water piped**	**40 (54.1)**	**28 (82.4)**	**0.01**
** HH Flush toilet**	**9 (12.2)**	**12 (35.3)**	**0.01**
** HH Tile floor**	**19 (25.7)**	**18 (52.9)**	**0.01**
**HH Livestock**	23 (31.1)	17 (48.6)	0.09
**HH Agriculture**	14 (18.9)	6 (17.1)	1.00
**HH daily food expenditure (USD)** ^e^ **, mean (range)**	**3.0 (0–14.5)**	**4.7 (0–12.1)**	**0.01** **
**Prior food aid**	**35 (47.3)**	**7 (20.0)**	**0.01**

^a^Severely food insecure: HFIAS score = 4, Not severely food insecure: HFIAS score 1–3; BMI = Body Mass Index; MUAC = Mid-upper arm circumference; IDDS = Individual Dietary Diversity Scale (see text); HH = Household

^b^Educated is defined as completed primary school or more

^c^Number of individuals per household

^d^Estimated household monthly income in U.S. Dollars

^e^Estimated U.S. Dollars spent on food for the household per day

*p-value for trend

**Mean and range based on raw data, p-value based on square root transformation

Malnutrition was associated with smaller MUAC, younger age, and higher educational level (Table 4). Malnutrition was not associated with dietary diversity, household size, economic indicators, livestock ownership, household agriculture, daily expenditure on household food, receipt of food aid, decreasing meal size, skipping meals, or spending 24 hours without eating as a result of insufficient food.

**Table 4:** Comparison of participant characteristics according to nutritional status.

	Malnourished^a^ (%)	Not malnourished (%)	p-value
**N**	25	84	**-**
**Food Insecure** ^b^	19 (76.0)	76 (90.5)	0.09
**Severely Food Insecure** ^b^	16 (64.0)	58 (69.0)	0.63
**MUAC, mean (range)**	**211 (145–245)**	**288 (225–385)**	**0.01**
** MUAC ≤ 230mm**	**21 (84.0)**	**3 (3.6)**	**0.01**
**IDDS score, mean**	6.44	6.14	0.49
**Age (years), mean (range)**	**38.6 (20–67)**	**45.1 (19–64)**	**0.01**
**Female**	18 (72.0)	73 (86.9)	0.12
**Marital status- Never married**	5 (20.0)	8 (9.5)	0.17
**Marital status- Widowed**	6 (24.0)	32 (38.1)	0.24
**Educated** ^c^	**12 (48.0)**	**19 (22.6)**	**0.02**
**Unemployed**	12 (48.0)	45 (53.6)	0.66
**Personal cell-phone**	18 (72.0)	68 (81.0)	0.40
**HH size** ^d^ **≤ 5**	7 (28.0)	24 (28.6)	1.00
**HH size** ^d^ **≤ 10**	16 (64.0)	58 (69.0)	0.63
**HH monthly income (USD)** ^e^ **, mean (range)**	98.7 (0–484.7)	144.8 (0–670.4)	0.24*
**Economic Indicators**			
** HH Electricity**	22 (88.0)	58 (69.9)	0.12
** HH Television**	20 (80.0)	50 (60.2)	0.10
** HH Water piped**	16 (64.0)	52 (62.7)	1.00
** HH Flush toilet**	5 (20.0)	16 (19.3)	1.00
** HH Tile floor**	9 (36.0)	28 (33.7)	0.82
**HH Livestock**	9 (36.0)	31 (36.9)	1.00
**HH Agriculture**	3 (12.0)	17 (20.2)	0.56
**HH daily food expenditure (USD)** ^f^ **, mean (range)**	2.9 (0–9.7)	3.7 (0–14.5)	0.13*
**Prior food aid**	13 (52.0)	29 (34.5)	0.16
**Decrease meal size due to food insecurity**	12 (48.0)	59 (70.2)	0.06
**Skip meals due to food insecurity**	11 (44.0)	40 (47.6)	0.82
**Remain 24 hours without eating due to food insecurity**	2 (8.0)	12 (14.3)	0.52

^a^Malnourished = BMI <18.5

^b^Food Insecure: HFIAS = 2–4, Severely Food Insecure: HFIAS score = 4; MUAC = Mid-upper arm circumference; IDDS = Individual Dietary Diversity Scale (see text); HH = Household

^c^Educated is defined as completed primary school or more

^d^Number of individuals per household

^e^Estimated household monthly income in U.S. Dollars

^f^Estimated U.S. Dollars spent on food for the household per day

*Mean and range based on raw data, p-value based on square root transformation.

Severe food insecurity was not associated with CD4 count or stage 3 or 4 disease (Table 5). However, severe food insecurity was associated with missing appointments due to hunger (p = 0.01) and not taking ART due to hunger (p = 0.02). There was no association between severe food insecurity and the number of days in which participants failed to take ART in the past week. Malnutrition was associated with lower CD4 counts (p = 0.01). Malnutrition was not associated with stage 3 or 4 disease, missing appointments due to hunger or not taking ART due to hunger. Among those who were malnourished 35.0% failed to take their ART at least once in the past week versus approximately 17.0% of those who were not malnourished (p = 0.12).

**Table 5:** Comparison of HIV outcomes and associated behaviors according to food security status and nutritional status.

	**Mean CD4**	**WHO Stage 3 or 4 n (%)**	**Miss appointments due to hunger n (%)**	**Don’t take ART due to hunger n (%)**	**Miss meals due to ART effects n (%)**	**Missed any ART in the past 7 days n (%)**
**Severely food insecure** ^a^ **(N = 74)**	412	32 (50.0)	**12 (16.9)**	**15 (23.1)**	42 (64.6)	14 (21.5)
**Not severely food insecure (N = 35)**	457	19 (63.3)	**0 (0)**	**1 (3.2)**	17 (54.8)	6 (19.4)
**p-value**	0.48	0.27	**0.01**	**0.02**	0.38	1.00
**Malnourished** ^b^ **(N = 25)**	**277**	10 (52.6)	3 (13.0)	4 (20.0)	14 (70.0)	7 (35.0)
**Not malnourished (N = 84)**	**470**	41 (54.7)	9 (11.0)	12 (15.8)	45 (59.2)	13 (17.1)
**p-value**	**0.01**	1.00	0.72	0.74	0.45	0.12
	**Mean CD4**	**WHO Stage 3 or 4 n (%)**	**Miss appointments due to hunger n (%)**	**Don’t take ART due to hunger n (%)**	**Miss meals due to ART effects n (%)**	**Missed any ART in the past 7 days n (%)**
**Severely food insecure** ^a^ **(N = 74)**	412	32 (50.0)	**12 (16.9)**	**15 (23.1)**	42 (64.6)	14 (21.5)
**Not severely food insecure (N = 35)**	457	19 (63.3)	**0 (0)**	**1 (3.2)**	17 (54.8)	6 (19.4)
**p-value**	0.48	0.27	**0.01**	**0.02**	0.38	1.00
**Malnourished** ^b^ **(N = 25)**	**277**	10 (52.6)	3 (13.0)	4 (20.0)	14 (70.0)	7 (35.0)
**Not malnourished (N = 84)**	**470**	41 (54.7)	9 (11.0)	12 (15.8)	45 (59.2)	13 (17.1)
**p-value**	**0.01**	1.00	0.72	0.74	0.45	0.12

^a^Severely food insecure: HFIAS score = 4; Not severely food insecure: HFIAS score 1–3.

^b^Malnourished = BMI <18.5

Lower BMI was associated with younger age and lower CD4 counts when controlling for years since HIV diagnosis and years on ART (Table 6).

**Table 6:** Association between age and CD4 count and BMI, when controlling for years on ART and years since HIV diagnosis.

	Simple linear regression			Multiple linear regression^a^		
	Beta	CI (95%)	p-value	Beta	CI (95%)	p-value
Age	0.150	0.053–0.247	**0.01**	0.105	0.012–0.197	**0.03**
CD4	0.007	0.003–0.010	**0.01**	0.007	0.004–0.011	**0.01**
Years on ART^a^	-0.088	-0.398–0.221	0.57	-0.098	-0.653–0.457	0.73
Years since HIV diagnosis	0.132	-0.081–0.345	0.22	-0.283	-0.713–0.147	0.19

^a^n = 89. Individuals not on ART or those with missing data were excluded.

## Discussion

In this study of food insecurity and malnutrition among HIV-infected adults in Senegal, three important findings emerged: (1) we found a high prevalence of severe food insecurity among HIV-infected adults in both Dakar and Ziguinchor, Senegal, (2) we found a high prevalence of malnutrition among HIV-infected adults on ART in Senegal, and (3) we found significant differences and unexpected similarities in both socioeconomic and HIV-associated characteristics between sites.

Our study demonstrates that the vast majority of HIV-infected adults in Dakar and Ziguinchor suffer from food insecurity. Furthermore, we have shown that the majority of individuals suffer not only from food insecurity, but severe food insecurity. In the urban capital of Dakar, which is the wealthiest region in the country [19], approximately 85% of participants were food insecure and 60% were severely food insecure. In Ziguinchor, approximately 90% of participants were food insecure and 75% were severely food insecure. As a result of food insecurity, the majority of individuals decrease meal size in order to have enough food for all members of the household and almost half skip meals. Many are forced to remain without eating for 24 hours or more because they do not have enough food.

Few studies have specifically quantified the prevalence of food insecurity among HIV-infected adults in sub-Saharan Africa. There have been two studies documenting the prevalence of food insecurity among HIV-infected adults in urban settings. In a study among 898 individuals on ART in Kinshasa, Democratic Republic of the Congo, 57% of individuals were food insecure and 50.9% were severely food insecure [13]. In Windhoek, Namibia, among 390 individuals on ART, 92% were food insecure and 67% were severely food insecure [14]. Studies among HIV-infected adults in rural settings have been conducted in Kenya [25, 26], Uganda [15, 27], and Ethiopia [28]. Using both the HFIAS and social worker assessments among 67,038 HIV-infected individuals across 17 sites in Western Kenya, the prevalence of food insecurity ranged from 20–50% [25]. In a second study conducted among 67 HIV-infected individuals living in a rural Kenyan community on an island in Lake Victoria, all were found to be food insecure and 79.1% were severely food insecure [26]. In rural Uganda, the prevalence of food insecurity among 456 HIV-infected adults starting ART was 74.5%, with a prevalence of severe food insecurity of 37.9% [27]. In Northern Ethiopia, the prevalence of food insecurity among 376 HIV-infected adults was 40.4% [28]. Based on the results of our study, the prevalence of food insecurity and severe food insecurity among HIV-infected adults in Senegal is among the highest reported for similar populations in sub-Saharan Africa.

We did not find an association between severe food insecurity and malnutrition or BMI. Severe food insecurity was associated with less education and lower socioeconomic status. These findings are similar to those seen in studies evaluating food insecurity in general populations irrespective of HIV status, where educational level and poverty have been identified as determinants of food insecurity [29, 30]. Contrary to previous studies, we did not find an association between severe food insecurity and household size [26, 29–32]. This is likely a consequence of our limited sample size, as we did find a trend towards smaller household size among those who were not severely food insecure. We postulated that household livestock would be protective against severe food insecurity. There are numerous plausible mechanisms by which individuals who own livestock are less likely to be food insecure. While livestock can be used as a source of food, they additionally represent valuable assets that can be sold to increase household income [33]. Surprisingly, neither livestock ownership nor household agriculture were protective against severe food insecurity. This likely represents a need for deeper evaluation and larger sample size rather than a true lack of association.

In previous studies lower dietary diversity has been associated with greater mortality and worse clinical outcomes among HIV-infected adults [34, 35]. Out of a maximum possible score of 12 food groups, our participants had a mean Individual Dietary Diversity Scale (IDDS) score of 6.2. Despite greater household agriculture and livestock ownership in Ziguinchor, the mean IDDS score did not differ between sites. Dietary diversity has been used as a measure of food security [36, 37] and lower dietary diversity has been associated with food insecurity among people living with HIV [32]. This is consistent with our finding that severe food insecurity was associated with lower dietary diversity.

Our assessment of ART adherence was limited in that we did not review pharmacy refill records or calculate the medication possession ratio (MPR). Nonetheless, based on the results of participant interviews we found that among HIV-infected adults in Senegal, severe food insecurity is associated with lower attendance at clinic appointments and lower adherence to antiretroviral therapy. Among severely food insecure individuals, approximately 23% reported not taking their ART due to hunger and approximately 17% reported missing clinic appointments due to hunger. In interviews conducted with HIV-infected patients in Rwanda [38], Uganda [39, 40], Tanzania [39], Botswana [39], South Africa [41], Cameroon [42], Zambia [43, 44], Kenya [26], Ethiopia [45], and Democratic Republic of Congo (DRC) [46], food insecurity was consistently reported by patients as a barrier to ART adherence. In the DRC [13] and Namibia [14], food insecurity was associated with increased odds of poor adherence to ART (AOR 2.06 and OR 3.84) as measured by the MPR. In a longitudinal study conducted in Uganda, food insecurity was associated with poor ART adherence (AOR = 1.56), incomplete virologic suppression (AOR = 1.52), and a CD4 count <350 (AOR = 1.47) [15]. In addition, we found that overall, ART adherence was poor, with approximately 21% of all patients reporting missed ART doses in the past 7 days. These findings have important implications for the success of HIV treatment programs in Senegal and warrant further evaluation.

We found a high prevalence of malnutrition among HIV-infected adults on ART in Senegal. Approximately one fifth of HIV-infected adults in Dakar were malnourished and slightly more than a quarter of HIV-infected adults in Ziguinchor were malnourished. The overall prevalence of very severe underweight (BMI <15) was approximately 4%. MUAC ≤230mm has been used as a rapid measure of malnutrition, especially in resource-limited settings [24] and low MUAC is associated with increased mortality among HIV-infected adults [10, 47]. In Dakar, approximately 23% of individuals had a MUAC ≤230mm, which was slightly higher than in Ziguinchor. In our study, the vast majority of participants were receiving ART. Numerous studies conducted in sub-Saharan Africa provide the prevalence of malnutrition among HIV-infected adults starting ART [2–5, 7, 8, 10, 11, 48, 49], but few provide the prevalence of malnutrition following multiple years on ART. In a meta-analysis using pooled DHS data from 11 countries in sub-Saharan Africa, the prevalence of malnutrition (BMI <18.5) among HIV-infected women was 10.3%, however, ART status was not specified [50]. In Ethiopia, among 376 HIV-infected women on ART for a mean of 3.4 years, the prevalence of malnutrition (BMI <18.5) was 42.3% [28]. Interpreting the results of our study in the context of the Integrated Food Security Phase Classification System established by the U.N. FAO [51], HIV-infected adults in Senegal are at Phase 3 “Crisis” level.

Unlike food insecurity, malnutrition was not associated with socioeconomic factors or lower educational level. This finding is inconsistent with previous studies in which malnutrition has been associated with lower socioeconomic status, lower educational level, and unemployment [52–55] and may be a consequence of our limited sample size. We found that lower BMI was associated with younger age. This finding is consistent with studies of malnutrition among non-HIV infected populations [53, 54]. Contrary to our expectations, household agriculture and livestock were not protective against malnutrition. Agriculture practices and livestock use should be further evaluated in order to better characterize these relationships.

The association between malnutrition and mortality among individuals starting ART has been well documented [1–11]. Malnutrition has additionally been associated with advanced WHO stage [52] and poor adherence [45]. In this cross-sectional study we explored potential associations between malnutrition, BMI, and HIV measures and outcomes, including time since diagnosis, WHO stage, time on ART, ART regimen, adherence, and CD4 count. We found that malnutrition was associated with lower CD4 counts. We also found that among individuals receiving ART, lower BMI was associated with lower CD4 counts but not time since HIV diagnosis or time on ART. This finding is of particular interest given that the majority of participants had been receiving ART for multiple years. This suggests either non-HIV associated etiologies of malnutrition or HIV-associated etiologies resulting from ART adverse effects, poor adherence, or treatment failure, rather than untreated HIV. The majority of patients reported missing meals due to ART effects, irrespective of nutritional status, and nutritional status did not differ according to ART regimen. If poor adherence or treatment failure are contributing to malnutrition and low BMI, we would expect to see an association between malnutrition and virologic failure. Future studies, which include viral load monitoring, would help to clarify the mechanism by which malnutrition and low BMI are associated with low CD4 counts among individuals receiving ART in Senegal.

We found significant differences and unexpected similarities in both socioeconomic and HIV-associated characteristics between sites. Dakar is the country’s capital and the wealthiest region in the country. Ziguinchor is located in the Casamance region, which recently emerged from a decades long civil war, leaving formerly arable land violated by land mines. Based on household wealth indicators, participants in Ziguinchor were of lower socioeconomic status. This is consistent with differences in household daily food expenditure, where individuals in Dakar spent more than twice that of individuals in Ziguinchor. However, despite greater spending in Dakar, food insecurity and malnutrition were prevalent in both sites. Understanding potential differences in purchasing power in Dakar compared to Ziguinchor is of critical importance in calculating the cost of interventions in each site.

The mean transport time to clinic in Dakar was more than twice that of the transport time in Ziguinchor. While the majority of individuals reported living locally in either Dakar or Ziguinchor, it is possible that subjects in Dakar live further from clinic and that traffic in Dakar contributed to increased transport time. These differences should be taken into account when planning potential clinic based interventions, as both transport time and costs are known barriers to care [39, 56, 57].

We expected that individuals in Dakar would have higher levels of education and employment compared to those in Ziguinchor; however, educational level and employment status did not differ between sites. Overall, less than a third of participants were educated beyond primary school and approximately half were unemployed. While household size did not differ between sites, there was a difference in marital status. In Ziguinchor, a greater proportion of individuals were never married, whereas in Dakar there were a greater number of widows. Overall, livestock ownership was more common than practicing agriculture. In Ziguinchor, approximately half of individuals owned livestock and a third practiced agriculture. Even in urban Dakar, almost a quarter of individuals owned livestock. A deeper understanding of the role of agriculture and livestock in the household could greatly inform potential intervention efforts.

Previous studies have documented a higher prevalence of HIV-2 in Ziguinchor compared to Dakar [58]. In our study, 23.5% of participants in Ziguinchor were infected with HIV-2 and 3.9% were dually infected, compared to 15.7% infected with HIV-2 in Dakar. The majority of individuals at both sites were receiving ART. We found that although time on ART did not differ between sites, individuals in Ziguinchor had lower mean CD4 cell counts compared to Dakar.

Our study had several limitations. Due to the cross sectional study design we were unable to evaluate how the relationships between HIV, food insecurity, and malnutrition change over time. We used chart review to determine HIV measures and outcomes, which was subject to incomplete data bias. Our assessment of adherence is likely an underestimate, as we used patient reported measures of adherence and did not review pharmacy refill records. This study was conducted in a resource limited setting with minimal laboratory monitoring, therefore we were unable to determine if there is an association between food insecurity and virologic failure or ART resistance. We used anthropometric measures of malnutrition, thus we did not evaluate malnutrition associated with micronutrient deficiencies. Because our study was conducted in outpatient clinics, it likely does not capture individuals who are least adherent or individuals who are too ill to attend clinic appointments. This study was conducted during February and March, therefore it does not capture the influence of seasonality on food insecurity and malnutrition. The prevalence of food insecurity and malnutrition would be expected to be higher during the lean season, which lasts from June to September in Senegal and corresponds to the period between harvests. The majority of participants in our study were women. While the prevalence of HIV in Senegal is higher among women compared to men [21], our study may not be representative due to the disproportionate representation of women. Finally, our findings were additionally limited by small sample size and may not be representative due to the exclusion of pregnant women and children.

## Conclusion

The findings of this study warrant further evaluation and urgent interventions to improve food security and reduce malnutrition. This is the first study to document the high prevalence not only of food insecurity, but severe food insecurity, among HIV-infected adults in both Dakar and Ziguinchor, Senegal. This is also the first study to document the high prevalence of malnutrition among HIV-infected adults on ART in Senegal. We found that severe food insecurity is associated with lower attendance at clinic appointments and lower adherence to antiretroviral therapy, and that malnutrition is associated with lower CD4 cell counts. Despite significant differences in socioeconomic and HIV-associated factors between sites, we found that severe food insecurity and malnutrition are the tragic norm for HIV-infected adults in both Dakar and Ziguinchor. These differences could potentially contribute to differing etiologies of food insecurity and malnutrition and suggest a need for site specific interventions.
